# Clinical outcome and toxicity of radiotherapy for inferior vena cava tumor thrombus in HCC patients: A retrospective study

**DOI:** 10.1097/MD.0000000000026390

**Published:** 2021-06-25

**Authors:** So Jung Lee, Hong Seok Jang, Yoo Kyung Choi

**Affiliations:** Department of Radiation Oncology, Seoul St. Mary's Hospital, College of Medicine, The Catholic University of Korea, Seoul, Korea.

**Keywords:** Hepatocellular carcinoma, inferior vena cava tumor thrombus, radiotherapy, toxicity

## Abstract

Hepatocellular carcinoma (HCC) involving the inferior vena cava rarely occurs, but its prognosis is extremely poor, with no established treatment to date. This study aimed to analyze the clinical outcome and toxicity of radiotherapy (RT) targeting inferior vena cava tumor thrombus (IVCTT) in HCC patients.

From November 2011 to July 2020, medical record of 19 HCC patients who were treated with RT for IVCTT was retrospectively reviewed. RT was delivered using 3-dimensional conformal radiation therapy, intensity-modulated radiation therapy, and stereotactic body radiation therapy. The median radiation dose was 50 Gy (range, 45–55.8 Gy) for intensity-modulated radiation therapy and three-dimensional conformal radiotherapy. Stereotactic body radiation therapy was performed in 5 patients, for a total of 32 Gy in 4 fractions.

The median follow-up duration was 8.1 months (range, 3.3–26.5 months). The median overall survival was 9.4 months (range, 3.7–26.5 months), and the 1-year overall survival rate was 37.1%. Eight of 19 patients (42.1%) had extrahepatic metastasis at the start of RT. Six of 11 patients (54.5%) who did not have extrahepatic metastasis at the start of RT showed extrahepatic metastasis after RT. The major cause of death was progression of extrahepatic metastasis (11 patients, 57.9%). The overall response rate of IVCTT for RT was 84.2%, and the local control rate at the time of the last follow-up was 89.4%. After RT, the most common first progression site was the lungs (9 patients, 47.4%). Most toxicities were grade 1 to 2 gastrointestinal (26.3%) and liver enzyme elevation (68.4%). Three patients occurred pulmonary embolism after RT later than 5 months after.

RT is a feasible and safe local therapy for IVCTT, with favorable tumor control and acceptable toxicity. Extrahepatic metastasis is the major progression pattern and a leading cause of death in patients treated with RT. The combination of effective systemic therapy with RT may have to be considered.

## 1 Introduction

Inferior vena cava (IVC) invasion of hepatocellular carcinoma (HCC) is rare, accounting for 4% of HCC patients observed at diagnosis or during chemoembolization.^[1]^ The prognosis of HCC patients with inferior vena cava tumor thrombus (IVCTT) is extremely poor, with the median survival of untreated patients being 2 to 5 months.^[2,3]^ IVCTT can be a source of lung metastasis and can lead to fatal complications such as pulmonary embolism and heart failure.^[4,5]^ Surgery, transarterial chemoembolization (TACE), radiotherapy (RT), and systemic agents such as sorafenib have been attempted as treatment options (5–8), no standard treatment has been established yet. Since RT is a noninvasive treatment and is not restricted by the tumor site, it has been attempted as a local therapy for IVCTT, showing relatively good response rate and local control.^[6–10]^ In a recent meta-analysis of RT for IVCTT, an overall response rate of 59.2%, local control rate of 83.8%, and 1-year overall survival rate of 53.6% have been reported.^[10]^ RT for IVCTT shows good local control rate, at 80% to 90% at the treatment site, but the median overall survival of patients who received RT was only 6 to 10 months.^[6,7,9,11]^ This study aimed to analyze the clinical outcome of RT for IVCTT by investigating the disease progression pattern and cause of death after RT, as well as local control. In addition, we tried to evaluate the toxicity of RT, specifically, the occurrence of pulmonary embolism, a critical complication that can be caused by dislodgement of tumor thrombus during RT.^[10]^

## 2 Materials and methods

### 2.1 Patients

Out of 20 patients who received RT targeting IVCTT from November 2011 to July 2020, except 1 patient who expired during the RT, 19 patients were retrospectively reviewed. In 19 patients, IVCTT was accompanied during treatment of HCC or upon recurrence of HCC. RT was performed for local control of IVCTT or to relieve symptoms caused by IVCTT such as abdominal distension or leg edema. The indications of patients included in this study were as follows. HCC was diagnosed through histological examination or the imaging criteria of the Korean Live Cancer Study group.^[12]^ The IVCTT was diagnosed by characteristic findings such as the presence of thrombus enhancement or diffusion restriction, evaluated through dynamic liver computed tomography (CT) or Primovist liver dynamic magnetic resonance imaging (MRI). Patients with Eastern Cooperative Oncology Group performance status ≤2. Patients with a normal liver volume (whole liver volume–target volume) of 800 cc or more. The patients who did not complete RT were excluded from the study. This study was conducted with ethical approval of the Institutional Review Board of the Catholic Medical Center (IRB No.: KC21RASI0037).

### 2.2 Radiotherapy

RT was performed using the intensity-modulated radiotherapy (IMRT), three-dimensional conformal radiotherapy (3D-CRT), and stereotactic body radiotherapy (SBRT) techniques in 12, 2, and 5 patients, respectively. RT was delivered using linear accelerators or helical tomotherapy (HT Accuray, Sunnyvale, CA). All the patients were immobilized by a customized device in the supine position with both arms above the head during simulation. Simulation was performed with an enhanced 4-dimensional computed tomography to evaluate target and organ movement under 3 mm slices using a LightSpeed RT 16 CT scanner (GE Healthcare Inc, Waukesha, WI). All the patients received RT targeting IVCTT. Four patients were treated with RT including the right atrium tumor thrombus. Three patients received RT including intrahepatic lesions and another 3 patients including portal vein tumor thrombus (PVTT). The gross tumor volume (GTV) was delineated by referring to the dynamic liver CT and the Primovist dynamic MRI. Considering the internal target motion, internal target volume was set with a 5 to 15 mm margin from the GTV, and the planning target volume was set with a 0 to 5 mm margin from the internal target volume. For IMRT or 3D-CRT planning, the mean liver dose was restricted at ≤28 Gy, and the V30 (the portion irradiated with ≥30 Gy) of the normal liver was less than 40%. For SBRT, the mean liver dose was restricted to <15 Gy, the normal liver volume irradiated with <15 Gy was ≥800 cc, and the maximum permissible dose to the stomach and duodenum was 27 Gy.

### 2.3 Assessment and follow-up

The first follow-up was performed–1 to 2 months after RT completion, followed by 3 to 4 month intervals. At follow-up, evaluation of performance status and physical examination, complete blood count, liver function test, and liver CT or MRI were conducted. Treatment response was evaluated according to the Response Evaluation Criteria In Solid Tumors (RECIST) 1.1 through liver CT or MRI. The overall response was defined as a case showing complete response (CR) or partial response (PR). Local control was defined as being maintained without progression of the lesion in the RT field. Local progression-free survival was defined as the duration between the start date of RT and the date of occurrence of local progression in the RT field or the date of last follow-up or death. The first progression site was defined as the site showing the disease progression at first after RT, including the development of extrahepatic metastasis or progression of existing lesion. Toxicity was evaluated for gastrointestinal and hepatic system up to 3 months after RT completion by Common Terminology Criteria for Adverse Events (version 5.0). The patient's ascites, liver function test (aspartate transaminase, alanine aminotransferase, bilirubin, albumin), and Child-Puch score were evaluated. In the evaluation of gastrointestinal toxicity, history taking was performed for nausea, dyspepsia, esophagitis, or gastritis symptom. In the case of grade 2 or higher gastrointestinal symptom, esophagogastroduodenoscopy was performed.

### 2.4 Statistical analyses

The actuarial overall survival was calculated using the Kaplan–Meier method, and the significance of prognostic factors with survival was analyzed using the log-rank test. OS was defined as the duration between the start date of RT and the date of death or last follow-up. A *P* value <0.05 was considered statistically significant. Statistical analyses were performed using R version 4.0.0 for Windows (R Development Core Team, Vienna, Austria).

## 3 Results

### 3.1 Patient and treatment characteristics

The median follow-up duration was 8.1 months (range, 3.3–26.5 months), and the patient's median age was 58 years (range, 44–78 years). The majority of patients (14 patients, 73.7%) were Child–Pugh classification A at RT initiation, and only 1 patient had Child–Pugh classification C. Seven patients (36.8%) had tumor thrombus extensions to the right atrium, and 10 patients had accompanying PVTTs. At the start of RT, 8 patients (42.1%) had extrahepatic metastases and 3 (15.8%) had lymph node metastases. Details of the patient characteristics are shown in Table 1.

**Table 1:** Patients and tumor characteristics.

Factor	No. (%) (total = 19)
Age (years)	Median, 58 (range, 44–78 yrs)
Gender	
Male	16 (84.2)
Female	3 (15.8)
ECOG-performance status	
0	4 (21.1)
1	12 (63.1)
2	3 (15.8)
Child–Puch classification	
A	14 (73.7)
B	4 (21.0)
C	1 (5.3)
Etiology of chronic liver disease	
HBV	17 (89.4)
HCV	1 (5.3)
NBNC	1 (5.3)
Intrahepatic tumor number	
No viable portion	3 (15.8)
Solitary	3 (15.8)
Multiple	13 (68.4)
Intrahepatic tumor size (largest diameter)	
<5 cm	8 (42.1)
≥ 5 cm and < 10 cm	6 (31.6)
≥ 10 cm	5 (26.3)
PVTT invasion	
Present	10 (52.6)
Absent	9 (47.4)
LN metastasis	
Present	3 (15.8)
Absent	16 (84.2)
Tumor thrombus extension to right. atrium	
Present	7 (36.8)
Absent	12 (63.2)
Extrahepatic metastasis	
Present	8 (42.1)
Absent	11 (57.9)

ECOG = Eastern Cooperative Oncology Group, HBV = hepatitis B virus, HCV = hepatitis C virus, LN = lymph node, NBNC = non-hepatitis B viral and non-hepatitis C viral, No. = number, PVTT = portal vein tumor thrombus.

The median GTV and planning target volumes were 32.05 cc (range, 5.6–363 cc) and 177 cc (range, 28.67–910.7 cc), respectively. The median radiation dose with an equivalent dose of 2 Gy per fraction with an a/b ratio of 10 (EQD_10/2_) was 50 Gy_10/2_ (range, 48.8–62.5 Gy_10/2_) for IMRT and 3D-CRT. SBRT was performed in 5 patients, with a total of 32 Gy in 4 fractions. Combination therapy was defined as the other treatment modality performed within 1 month before or after RT. In combination therapy, TACE and hepatic artery infusion chemotherapy (HAIC) were administered in 5 (26.3%) and 4 (21.1%) patients, respectively.

After RT completion, 5 patients (2.63%) did not receive any further treatment. Seven patients (36.8%) received TACE, and 2 patients received HAIC. Eleven (57.9%) of 19 patients received systemic therapy (systemic chemotherapy, 5 patients; sorafenib, 3 patients; both systemic chemotherapy and sorafenib, 3 patients) before and after RT. Details of the treatment characteristics are shown in Table 2.

**Table 2:** Treatment's characteristics.

Previous Tx.	No. (%)
None	2 (10.5)
Operation	3 (15.8)
Transplantation	2 (10.5)
TACE/TAE	12 (63.2)
TARE	2 (10.5)
RFA	5 (26.3)
Systemic treatment	
-Sorafenib	3 (15.8)
-Systemic CTx.	2 (10.5)
Combination Tx. With RT	
None	10 (31.6)
TACE	5 (26.3)
HAIC	4 (21.1)
Tx. after RT	
None	5 (26.3)
TACE	7 (36.8)
HAIC	2 (10.5)
Systemic treatment	8 (42.1)
-Sorafenib	5 (26.3)
-Systemic CTx.	3 (15.8)
RT Technique	
3D-CRT	2 (10.5)
IMRT	12 (63.2)
SBRT	5 (26.3)
RT field	
IVCTT	8 (42.1)
IVC-RA tumor thrombus	2 (10.5)
IVCTT+ mass	4 (21.1)
IVCTT+ PVTT	3 (15.8)
IVC-RA TT + PVTT	2 (10.5)
RT dose (EQD_2/10_, Gy)	
< 50	8 (42.1)
≥ 50	11 (57.9)

3D-CRT = 3-dimensional conformal radiotherapy, CTx. = chemotherapy, EQD = effective cumulative dose, Gy = gray, HAIC = hepatic artery infusion chemotherapy, IMRT = intensity-modulated radiation therapy, IVCTT = inferior vena cava tumor thrombus, PVTT = portal vein tumor thrombus, RA = right atrium, RFA = radiofrequency ablation, SBRT = stereotactic body radiation therapy, TACE = transarterial chemoembolization, TAE = transarterial embolization, TARE = transarterial radioembolization, TT = tumor thrombus, Tx. = therapy.

### 3.2 IVC response and local control

**Figure 1:**
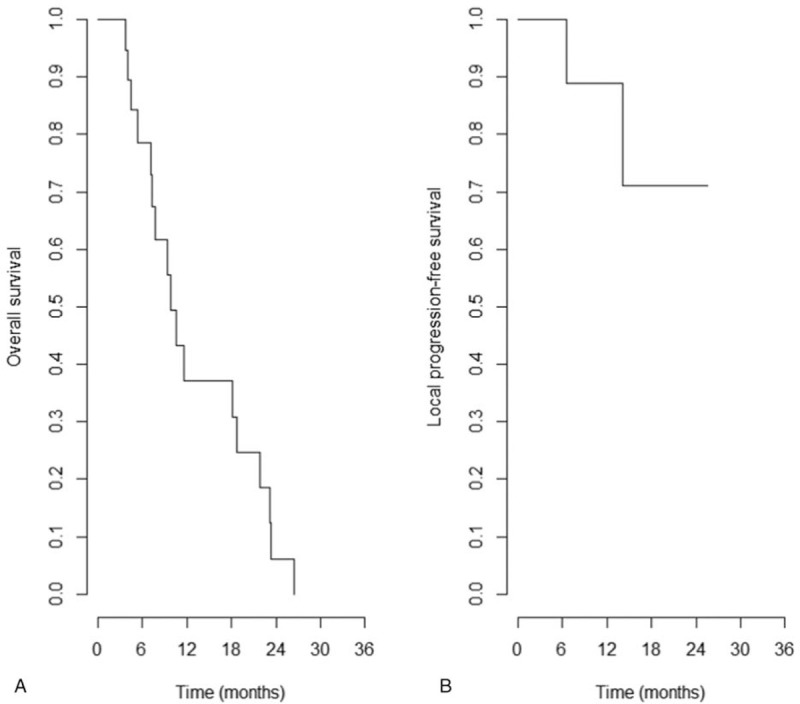
Kaplan–Meier graphs of overall survival (A) and local progression-free survival (B). One-year overall survival rate was 37.1% and 1-year local progression-free survival rate was 88.9%.

Sixteen patients (84.2%) showed an overall response. CR and PR were observed in 1 and 15 patients, respectively. No patient showed progressive disease, and 3 patients showed stable disease. The local control rate at the last analysis was 89.4%. The 1-year local progression-free survival rate was 88.9% (Fig. 1). During follow-up, only 3 patients showed progression within the RT field. One patient showed progression in the PVTT lesion within the RT field, and 2 patients showed progression in the IVCTT.

### 3.3 Survival and pattern of failure

**Figure 2:**
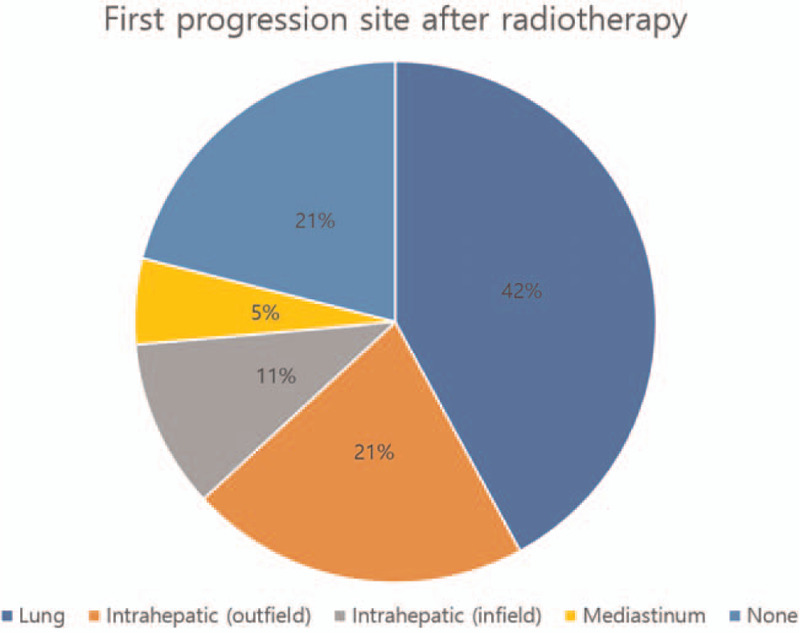
First progression site after radiotherapy. The most common first progression site after radiotherapy was lung (8 patients, 42.1%).

The median OS was 9.4 months (range, 3.7–26.5 months), and the 1-year OS rate was 37.1% (Fig. 1). Up to the last follow-up, a total of 17 patients died. Ten patients (52.6%) expired due to progression of extrahepatic metastasis and 4 due to progression of intrahepatic lesion. One patient died of tension pneumothorax. The causes of death of the 2 other patients were unavailable from the medical records. The median OS of patients with and without extrahepatic metastasis at the start of RT were 8.6 ± 4.9 months and 14.3 ± 8.4 months, respectively. Six (54.5%) of 11 patients who did not have extrahepatic metastasis at the start of RT showed extrahepatic metastasis after RT. The median duration from the start of RT to the onset of extrahepatic metastasis was 7.2 months (range, 3.3–14.7 months). The initial extrahepatic metastatic site was all lung. Moreover, the most common first progression site after RT was lung (8 patients, 42.1%). The first progression in 6 patients occurred intrahepatically, of which 4 occurred outside of the RT field and 2 in the RT field. Disease progression did not occur in 4 patients until the last follow-up. The first progression sites were illustrated in Figure 2.

### 3.4 Prognostic factor for overall survival

In univariate analysis, multiple intrahepatic tumors showed significantly lower 1-year OS than single intrahepatic tumors (20% vs 80%, *P* = .003). Extrahepatic metastasis at the initiation of RT was a negative prognostic factor for 1-year OS (12.5% vs 58.9%, *P* = 0.02). The RT dose (EQD_10/2_ ≥ 50 Gy) did not show significant results for OS. In terms of the RT technique, SBRT showed unfavorable results for 1-year OS compared with 3D-CRT or IMRT (0% vs 50.5% vs 50%, *P* = 0.005). The results of univariate analysis were given in Table 3. In multivariate analysis, tumor number (solitary/multiple) and extrahepatic metastasis were still significant factors for OS (Table 4).

**Table 3:** Univariate analysis for overall survival.

Factor		No.	1-yr OS (%)	*P* value
Age (years)	≤ 58	10	22.5	.09
	> 58	9	53.3	
Tumor size	< 5 cm	8	45	.8
	≥ 5 cm and <10 cm	6	20.8	
	≥ 10 cm	5	13.6	
Tumor number	Solitary	6	80	.003^∗^
	Multiple	13	20	
LN metastasis	None	16	37.8	.5
	Present	3	33.3	
PVTT	None	9	38.9	.7
	Present	10	35.0	
CP_classification	A	14	39.7	1
	B or C	5	23.9	
Extrahepatic metastasis	None	11	58.9	.02^∗^
	Present	8	12.5	
Atrium extension	None	12	42.8	1
	Present	7	28.6	
Combination Tx.	without	6	22.2	.6
	With	13	42.3	
Systemic Tx.	without	8	0	.07
	With	11	63.6	
Sorafenib	without	11	20.2	.8
	With	8	46.9	
RT dose (EQD_2/10_)	< 50 Gy	8	37.5	.6
	≥ 50 Gy	11	35.4	
RT technique	3D-CRT	2	50.5	.005^∗^
	IMRT	12	50	
	SBRT	5	0	

3D-CRT = three-dimensional conformal radiotherapy, CP = Child–Puch, EQD = effective cumulative dose, IMRT = intensity-modulated radiation therapy, LN = lymph node, No. = number, OS = overall survival, PVTT = portal vein tumor thrombus, RT = radiotherapy, SBRT = stereotactic body radiation therapy, Tx. = therapy.

∗ Statistically significant.

**Table 4:** Multivariate analysis for overall survival.

Factor		HR (95% CI)	*P* value
Age	≤ 58/>58	2.47 (0.48–12.56)	.274
Tumor number	Solitary/multiple	28.07 (2.65–297.4)	.006^∗^
Extrahepatic metastasis	Without/with	9.54 (1.84–49.41)	.007^∗^
Systemic therapy	Without/with	0.54 (0.10–2.81)	.466
RT technique	3D-CRT/IMRT/SBRT	1.95 (0.82–4.59)	.125

3D-CRT = three-dimensional conformal radiotherapy, CI = confidence interval, HR = hazard ratio, IMRT = intensity-modulated radiation therapy, RT = radiotherapy, SBRT = stereotactic body radiation therapy.

∗ Statistically significant.

### 3.5 Toxicity

The most common toxicities were grade 1 to 2 hepatic toxicity. No grade 3 or higher toxicity was reported. Four patients had grade 1 dyspepsia and 1 patient showed grade 1 nausea. Regarding hepatic toxicity, 13 patients (68.4%) showed grade 1 to 2 elevation of AST or alanine aminotransferase. Two patients showed grade 3 elevation of liver enzyme, but this was related to TACE. Ten patients (52.6%) showed an elevation of bilirubin, 9 patients were grade 1 and 1 was grade 3. Two patients (10.5%) showed 2 or more elevated CP scores after 2 and 3 months after RT respectively, and thereafter showed a regression of the CP score.

### 3.6 Pulmonary thromboembolism

One of 19 patients had pulmonary thromboembolism (PTE) at the start of RT, it regressed after RT. Three (15.8%) of 18 patients who did not have PTE at the start of RT showed PTE after RT, occurring at 5, 7, and 18 months after RT, respectively. All 3 patients developed PTE along with lung metastasis and mediastinal node metastasis, and they were treated with 3D-CRT or IMRT. No patient developed PTE after SBRT.

## 4 Discussion

HCC invading the IVC has a very poor prognosis and is challenging, as there is no established treatment yet. RT has been tried as a local therapy for IVCTT.^[6–10]^ In this study, 19 HCC patients with IVCTT treated with RT showed an objective response of 84.2% and a local control rate of 89.4%. A recent meta-analysis by Rim et al for RT of IVCTT reported the pooled response rate of 59.2% and local control rate of 83.8%.^[10]^ The local control was similar to our study, but our reported response rate is superior to theirs. This is because the meta-analysis included several studies that evaluated the response using various criteria (RECIST, modified response evaluation criteria in solid tumor; mRECIST, and world health organization; WHO criteria). Also depending on the study, HCC mass and PVTT lesions were included in the RT field, which might have affected the response evaluation. Some retrospective studies also reported response rates of 70% to 80%, which are similar to our study.^[6,9,11]^

In this study, the median OS of the total cohort was 9.4 months (range, 3.7–26.5 months) and the 1-year OS rate was 37.1%. This is consistent with a previous study that reported a median OS of 6.6 to 10.1 months and a 1-year OS rate of 30% to 43.5%.^[4,9]^ The 1-year OS rate of our study was slightly lower than that reported in the meta-analysis by Rim et al, which was 53.6%,^[10]^ and this is due to 8 patients (42.1%) who had extrahepatic metastasis at the start of RT. In our study, extrahepatic metastasis at the start of RT was a statistically significant negative prognostic factor for OS. The 6 (54.5%) of 11 patients who did not have extrahepatic metastasis at the start of RT occurred extrahepatic metastasis after RT, and with the first metastatic site was lung in all patients To date, no studies have directly compared the incidence of lung metastasis in IVCTT patients who underwent RT and those who did not. Pao et al^[4]^ reported that patients who showed a response (CR or PR) to RT for IVCTT had better lung-metastasis-free survival than patients who showed no response to RT. Because of the limitations of a few patients and the inclusion of patients who had lung metastasis at the start of RT, statistical analysis for lung metastasis could not be performed in our study. RT is thought to have the effect of inhibiting lung metastasis through local control of the IVCTT lesion; however, whether it is possible to reduce lung metastasis incidence itself should be identified through further studies involving a larger number of patients.

In this study, systemic therapy, such as systemic chemotherapy or sorafenib, showed a tendency of improving OS, but it was not statistically significant. However, since the analysis was limited because of the small cohort size and patients who have extrahepatic metastasis at the start of RT, the effect of systemic therapy on the survival of patients who were treated with RT requires further study. In univariate analysis, the SBRT technique showed significantly lower OS than 3D-CRT or IMRT. It is probably because 2 of the 5 patients treated with SBRT had multiple extrahepatic metastases at the start of RT. This was not significant in the multivariate analysis.

Another major cause of death in our study was the intrahepatic lesion progression. Three patients who did not have a viable HCC lesion in the liver when IVCTT was diagnosed showed significantly longer survival (survived until last follow-up, 18.8 and 23 months, respectively). In addition, solitary HCC was a favorable factor for OS in our study. This is thought to be because a single lesion has a good prognosis itself, but also well-controlled intrahepatic lesion may prolong the survival. In patients with HCC invading the IVC, as with conventional HCC patients, effective local control for intrahepatic lesions is an important factor for survival, which has been suggested in a study by Hou et al.^[6]^

In terms of toxicity, most toxicities observed in our study were grade 1 to 2, which is consistent with other studies on RT for IVCTT.^[6–10]^ An increase in CP score of ≥2 was confirmed in 2 patients at 2 and 3 months after the end of RT, but thereafter decreased; hence, the likelihood of radiation-induced liver disease seemed to be low. Three cases of pulmonary embolism occurred after RT, but there was no case that seemed to be highly related to RT. Igaki et al presented 1 case of pulmonary embolism after RT on IVCTT, but it was reportedly difficult to assume that it is related to RT.^[7]^ Moreover, a study by Pao et al^[4]^ reported that among 36 patients who did not have pulmonary embolism before the start of RT, only 1 patient developed pulmonary embolism after RT. The authors mentioned that pulmonary embolism may be a natural course of the disease rather than a result of RT. Our study included 5 patients who underwent radiotherapy with SBRT, but no pulmonary embolism occurred in these patients. To date, there is no report that pulmonary embolism occurred when it was treated with SBRT.^[13–18]^ The occurrence of pulmonary embolism is not likely to be significantly correlated with the RT technique that uses a large fraction size; however, due to the limited number of patients in our study, this should be further clarified in future research.

Our study has several limitations. First, it has a relatively small number of patients and events. Therefore, some meaningful results might have been missed due to the limited number of patients. Second, as a retrospective study, various treatment strategies other than RT such as TACE or HAIC were combined. Survival may have been affected by these treatments. Third, since this study included patients treated with conventional fractionation or SBRT, there might be some difference in treatment effect according to the treatment technique. However, when considering the rarity of the disease, our study presented the clinical outcome that can be referenced through an analysis of failure patterns and the cause of death of patients who were treated with RT for IVCTT, and to find direction that further extends the survival of these patients. In addition, by analyzing the association between RT and toxicity, especially pulmonary embolism, we suggested that RT is a relatively safe local therapy without fatal complications.

## 5 Conclusion

In conclusion, our study showed that RT was a feasible and safe local therapy for IVCTT with favorable tumor control and acceptable toxicity. Extrahepatic metastasis was a major failure pattern and a leading cause of death in patients who were treated with RT. The combination of systemic treatment that is more effective for preventing extrahepatic metastasis with RT may need to be considered.

## Acknowledgments

The authors thank the reviewers for their thoughtful comments.

## Author contributions

**Conceptualization:** So Jung Lee, Hong Seok Jang

**Data curation:** So Jung Lee.

**Formal analysis:** So Jung Lee, Yoo Kyung Choi, Hong Seok Jang,

**Investigation:** So Jung Lee, Hong Seok Jang, Yoo Kyung Choi.

**Methodology:** So Jung Lee, Yoo Kyung Choi, Hong Seok Jang

**Supervision:** Hong Seok Jang

**Validation:** So Jung Lee

**Visualization:** So Jung Lee, Hong Seok Jang.

**Writing – original draft:** So Jung Lee

**Writing – review & editing:** So Jung Lee, Yoo Kyung Choi, Hong Seok Jang
